# Vitamin D pathway gene variation rs3740165 is associated with serological uric acid levels in healthy Chinese women

**DOI:** 10.3389/fendo.2022.1059964

**Published:** 2022-12-13

**Authors:** Jiemei Gu, Hua Yue, Chun Wang, Hao Zhang, Weiwei Hu, Zhenlin Zhang

**Affiliations:** Shanghai Clinical Research Center of Bone Disease, Department of Osteoporosis and Bone Diseases, Shanghai Sixth People’s Hospital Affiliated to Shanghai Jiao Tong University School of Medicine, Shanghai, China

**Keywords:** uric acid, vitamin D, pathway, polymorphism, gene

## Abstract

**Aim:**

To investigate the relationship between gene polymorphisms involved in the vitamin D metabolic pathway and serum uric acid (UA) levels in Chinese women.

**Methods:**

Fifteen key genes within the vitamin D metabolic pathway were screened using 96 single nucleotide polymorphisms in a group of 1,206 (37.96 ± 13.08 years) unrelated healthy Chinese women (aged 20–85 years). Blood and urine tests were performed at the same time. The Wilcoxon Mann–Whitney test was used to compare groups aged ≤50 years and >50 years. The mean serum UA values were computed within each group of homozygous referent, heterozygous, and homozygous variant genotypes for each single nucleotide polymorphism.

**Results:**

The exclusion process left 1,169 participants (38.16 ± 13.13 years) for analysis. One single nucleotide polymorphism in the *CUBN* gene (rs3740165) was identified as being significantly associated with serum UA levels in the group aged over 50 years. The wild type (C/C) population had higher serum UA levels in this group (P<0.001). In women aged over 50 years, allele C was associated with a higher risk of hyperuricemia than allele T (odds ratio 2.752, 95% confidence interval 1.458–5.192; P = 0.002). There was also a higher risk of hyperuricemia in genotype TC + CC compared with genotype TT (odds ratio 3.326, 95% confidence interval 1.513–7.313; P = 0.003) in women over 50 years of age.

**Conclusion:**

The results suggest that the *CUBN* gene contributes to variability in serum UA levels in healthy Chinese Han women over 50 years of age.

## 1 Introduction

Historically, serum uric acid (UA) has been known as a waste by-product that in excess may cause kidney stones and gouty arthritis (1, 2). It also seems to play an important role in multiple metabolic, hemodynamic, and homeostatic abnormalities, such as type 2 diabetes, cardiovascular disorders, hypertension, and dyslipidemia (3–6). Many studies indicate that UA is a key factor in the development of the metabolic syndrome (7–9).

Genetic research has demonstrated that UA levels are highly heritable (10). Three UA membrane transporters, *SLC22A12*, *SLC2A9*, and *ABCG2*, play key roles in the regulation of serum UA (11–16). About 30 gene variants have been identified, explaining nearly 7% of the variation in serum UA concentration by genome-wide association analyses (17). Thus, a large number of variations have not been explained.

Vitamin D deficiency is a major health problem worldwide. Over 1 billion individuals worldwide have been diagnosed with vitamin D deficiency or insufficiency (18), which causes both skeletal (19, 20) and extra-skeletal diseases (21, 22). Animal and human studies have suggested that the metabolic pathways of UA and vitamin D are related. In rats, induction of increased circulating UA suppresses 1α-hydroxylase, which leads to increased parathyroid hormone (PTH) levels and decreased 1α,25-dihydroxyvitamin D levels (22). Furthermore, in humans, administration of allopurinol reduces serum UA levels, which leads to a reduction in PTH levels and an increase in 1α,25-dihydroxyvitamin D levels (23, 24). Vitamin D insufficiency is significantly associated with elevated UA levels among postmenopausal Chinese Han women (24).

The *GC* (GC vitamin D binding protein) gene is an important gene in the vitamin D metabolic pathway (25, 26). Thakkinstian et al. suggested a potential causal association between the *GC* gene and UA through the 25-hydroxyvitamin D3 (25(OH)D) mediator (27). We, therefore, hypothesized that serum UA is associated with gene polymorphisms within the vitamin D metabolic pathway.

There are several genes within the vitamin D metabolic pathway, of which *CUBN* is one. *CUBN* encodes the protein cubilin, which is located in the epithelium of the intestine and kidneys. It acts as a co-transporter and helps in the uptake of lipoprotein, iron, and vitamins. In the proximal tubule cells, cubilin takes part in reabsorption of vitamin D binding protein from glomerular filtrates and assists in the synthesis of 1α,25-dihydroxyvitamin D3 (28, 29). *CUBN* consists of an amino terminal region involved in protein trimerization and membrane anchoring, followed by eight epidermal-growth-factor-type B repeats and 27 contiguous CUB (complement C1r/C1s, Uegf, BMP1) domains. *CUBN* has an amino terminal furin cleavage site, which is probably involved in proteolytic processing in the trans-Golgi and possibly in shedding the plasma-membrane-bound protein in the gut (30).

We screened 96 common variants of genes involved in the vitamin D metabolic pathway, which we reported previously (25), to explore the association between these gene variations and serum UA levels in 1,206 Chinese women.

## 2 Materials and methods

### 2.1 Study participants

A total of 1,518 Chinese woman living in Shanghai were recruited from several community centers. All participants were of Han ethnicity and in generally good health. The average menopausal age among 134,010 Chinese women was reported as 48.6 years old (31). Women’s serum UA levels increase after menopause; therefore, we chose the age of 50 years to divide participants into two groups.

We conducted a survey that provided the participants’ sex, age, and medical histories. After exclusion criteria were met, we recruited a total of 1,206 women (aged 20–85 years) with complete information. We excluded individuals with an estimated glomerular filtration rate <30 mL/min/1.73 m^2^ (32), hepatic disease, cancer, gout, hyperuricemia, myocardial infarction, heart failure, stroke, hypertensive encephalopathy, retinal hemorrhage, being pregnant, or any of the following medications that may alter serum urate (diuretic, allopurinol, corticosteroids, beta blockers, antituberculotics, quinolones, sulfonylureas, metformin, immunosuppressants, nicotinic acid, pancreatin, tumor chemotherapeutics, aspirin and levodopa). The study was approved by the Ethics Committee of the Shanghai Jiao Tong University Affiliated Sixth People’s Hospital. All participants signed informed consent forms before entering the study.

### 2.2 Laboratory measurements

We obtained fasting blood samples from the participants between 8:00 h and 10:00 h in the morning. Serum levels of calcium, phosphorus, and alkaline phosphatase were tested using a Hitachi 7600–020 automatic biochemistry analyzer. The intra-assay and inter-assay coefficients of variations (CVs) were 1.5% and 2.0% for calcium, 2.1% and 2.3% for phosphorus, and 2.5% and 4.5% for alkaline phosphatase. Serum creatinine was detected with the sarcosine oxidase-PAP method using the creatinine kit from the Shanghai KEHUA Bio-engineering Corporation (China). Serum UA was measured with an enzymatic method using the Pureauto SUA kit (Japan). The CV of detecting UA was lower than 5%. The linear range of UA is 11.896–4758.4 μmol/L. Total cholesterol, triglycerides, and glucose were measured using kits from Roche Diagnostics. The following measurements of biochemical markers were also made using electrochemiluminescence immunoassays from Roche Diagnostics: total P1NP kit for serum procollagen type 1 N-terminal propeptide (P1NP), β-crosslaps kit for β-cross-linked C-telopeptide of type 1 collagen (β-CTX), 25-hydroxyvitamin D3 kit for 25(OH)D, and intact PTH kit for PTH. The intra-assay and inter-assay CVs were 2.3% and 2.8% for P1NP, 2.5% and 3.5% for β-CTX, and 1.4% and 2.9% for PTH, respectively (26). The lower limit of detection of 25(OH)D was 4 ng/mL (10 mmol/L). The intra-assay CVs for 25(OH)D were 5.7% at the level of 25.2 ng/mL, 5.7% at the level of 39.9 ng/mL, and 5.4% at the level of 65.6 ng/mL. The inter-assay CVs for 25(OH)D were 9.9% at the level of 25.2 ng/mL, 7.3% at the level of 39.9 ng/mL, and 6.9% at the level of 65.6 ng/mL (25).

### 2.3 Candidate genes and tag SNP selection

Fifteen candidate genes were selected according to the following criteria (1): biological importance in vitamin D transportation, metabolism, or degradation; and (2) evidence of a significant association in a previous genome-wide association analysis. The selected genes were *CUBN*, *GC*, *CYP24A1*, *ACADSB*, *CYP2J2*, *NADSYN1*, *CYP2R1*, *CYP11A1*, *DHCR7*, *CYP2C9*, *PTH*, *CYP1A1*, *CYP3A4*, *CYP27A1*, and *CYP27B1*. The basic characteristics of these genes are shown in **Table 1**. The detailed functions of the candidate genes have been described previously (25). For the studied genes, tagging single nucleotide polymorphisms (SNPs) were selected from the International HapMap Project (http://www.hapmap.org/cgi-perl/gbrowse/hapmap3_B36), based on the criteria described in our former research (25).

**Table 1:** The basic characteristics of the 15 candidate genes.

Gene	Location	Full name	Selected SNPs
*CUBN*	10p12.31	Cubilin	15
*CYP24A1*	20q13	Vitamin D 24-hydroxylase	14
*GC*	4q12–q13	Vitamin D binding protein	14
*ACADSB*	10q26.13	Acyl-CoA dehydrogenase, short/branched chain	7
*CYP2J2*	1p31.3–p31.2	Cytochrome P450 2J2	6
*NADSYN1*	11q13.4	NAD synthetase 1	6
*CYP2R1*	11p15.2	Vitamin D 25-hydroxylase	5
*CYP11A1*	15q23–q24	Cytochrome P450 11A1	5
*DHCR7*	11q13.4	7-Dehydrocholesterol reductase	5
*CYP2C9*	10q24	Cytochrome P450 2C9	4
*PTH*	11p15.3–p15.1	Parathyroid hormone	4
*CYP1A1*	15q24.1	Cytochrome P450 1A1	3
*CYP3A4*	7q21.1	Cytochrome P450 3A4	3
*CYP27A1*	2q33–qter	Vitamin D (3) 25-hydroxylase	2
*CYP27B1*	12q13.1–q13.3	25 Hydroxyvitamin D-1-alpha hydroxylase	2

SNP, single nucleotide polymorphism; Acyl‐CoA, acetyl coenzyme A; AD, nicotinamide adenine dinucleotide.

### 2.4 SNP genotyping

Genomic DNA was extracted from peripheral blood samples and purified using routine methods. Genotyping was performed using the high-throughput Sequenom genotyping platform (MassARRAY matrix-assisted laser desorption/ionization-time of flight mass spectroscopy system; Sequenom, San Diego, CA, USA). Genotype frequencies were tested against the Hardy-Weinberg equilibrium using the chi-squared test.

### 2.5 Statistical analysis

Routine statistical analysis was performed using R software (https://www.r-project.org/). Baseline characteristics of participants in each age group were compared. The Kolmogorov–Smirnov test was used to evaluate data distribution for normality. All the data were skewed distributed; therefore, the Wilcoxon Mann–Whitney test was performed and all values were expressed as median and interquartile range. The mean serum UA values were computed within each group of homozygous referent, heterozygous, and homozygous variant genotypes for each SNP. A linear regression model was used to analyze the association between SNPs and serum UA. We used the LOWESS method to show the changes in serum UA in three different genotype carriers with age growth. The Bonferroni correction was used to adjust for multiple testing. The results were regarded as statistically significant at a value of P<0.05.

## 3 Results

**Figure 1:**
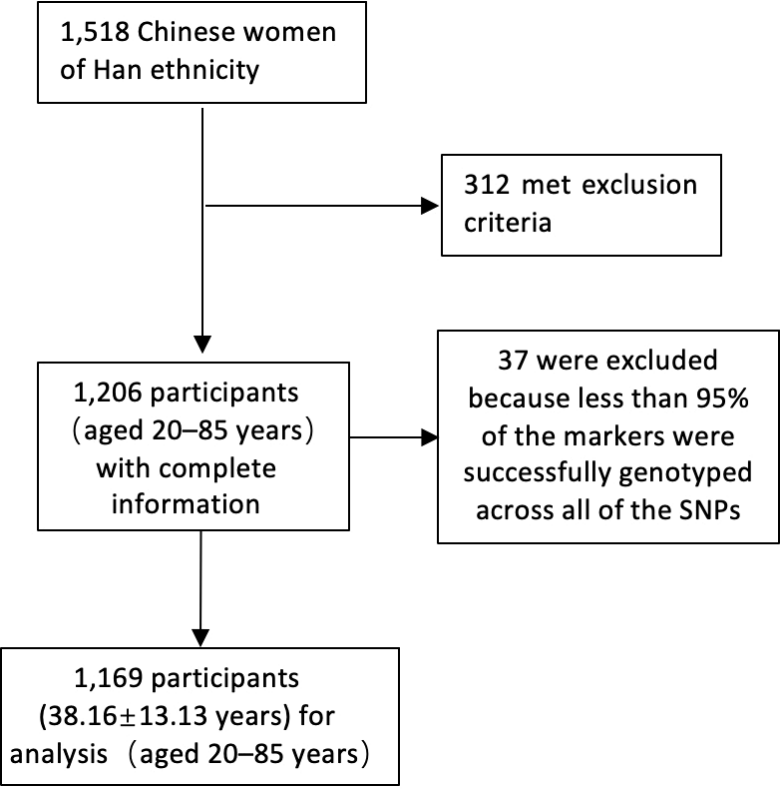
The inclusion process of the participants. SNP, single nucleotide polymorphism.

A total of 1,206 participants were recruited. Thirty-seven participants were excluded because less than 95% of the markers were successfully genotyped across all the SNPs. This exclusion process left 1,169 participants (38.16 ± 13.13 years) for analysis (aged 20–85 years). The inclusion process of the participants is shown in **Figure 1**. Among the women included in the current analysis, 940 (80.4%) were aged ≤50 years and 229 (19.6%) participants were aged over 50 years. The clinical characteristics and mean laboratory values of the 1,169 women are shown in **Table 2**. Ninety-five SNPs passed quality control criteria with genotyping call rates of greater than 90%. The basic characteristics of the SNPs are listed in the supplementary information (**Table S1**).

**Table 2:** Population characteristics and biochemical variables.

Characteristic/variable	All (n=1169)	Age ≤50 years (n=940)	Age >50 years (n=229)	P-value
Age (years)	35 (27–46)	31 (26–38)	55 (53–65)	<0.001
BMI (kg/m^2^)	21.34 (19.97–23.34)	21.19 (19.87–22.96)	23.44 (21.19–24.82)	<0.001
Uric acid (µmol/L)	253 (219–294)	251 (215–286)	266 (230–316)	<0.001
Calcium (µmol/L)	2.25 (2.17–2.36)	2.25(2.16–2.36)	2.26 (2.19–2.38)	0.05
Phosphorus (mmol/L)	1.1 (1–1.21)	1.1 (1.01–1.21)	1.1 (0.98–1.18)	0.136
ALP (U/L)	55 (46–65)	53 (45–63)	62 (51.5–78.5)	<0.001
Creatinine (µmol/L)	52 (47–59)	52 (47–58)	54 (48–63)	<0.001
eGFR (mL/min/1.73 m^2^)	117.5 (105.9–125.9)	121.1 (113.6–127.1)	100.1 (87.5–105.0)	<0.001
GLU (mmol/L)	4.49 (4.23–4.81)	4.48 (4.22–4.78)	4.57 (4.29–4.97)	0.002
TG (mmol/L)	0.76 (0.57–1.11)	0.72 (0.56–1.02)	0.93 (0.64–1.36)	<0.001
TC (mmol/L)	4.39 (3.85–4.93)	4.35 (3.82–4.85)	4.67 (3.98–5.22)	<0.001
P1NP (ng/L)	38.22 (29.91–52.28)	37.29 (29.23–49.70)	46.66 (32.04–60.23)	<0.001
β-CTX (ng/L)	290 (190–420)	270 (190–380)	420 (260–580)	<0.001
PTH (pg/mL)	34.19 (27.15–43.92)	34.36 (27.37–43.92)	33.66 (26.69–43.84)	0.474
25(OH)D (ng/mL)	20.03 (16.29–24.06)	20.1 (16.50–23.96)	19.6 (14.92–24.23)	0.417

All values are expressed as median and interquartile range. Wilcoxon Mann–Whitney test was used to compare the groups aged ≤50 years and >50 years. BMI, body mass index; ALP, alkaline phosphatase; eGFR, estimated glomerular filtration rate; GLU, glucose; TG, triglyceride; TC, total cholesterol; P1NP, procollagen type 1 N-terminal propeptide; β-CTX, β-cross-linked C-telopeptide of type 1 collagen; PTH, parathyroid hormone; 25(OH)D, 25-hydroxyvitamin D3.

Generally, women aged over 50 years had a higher body mass index and higher serum levels of UA, total cholesterol, triglycerides, P1NP, and β-CTX as well as a lower estimated glomerular filtration rate than women aged ≤50 years (P<0.001, **Table 2**). No association between biochemical markers including calcium, phosphorus, alkaline phosphatase, glucose, total cholesterol, triglycerides, PTH, 25(OH)D, P1NP, and β-CTX level with the studied polymorphisms was found.

One SNP in the *CUBN* gene (rs3740165) was identified as being significantly associated with serum UA in the group aged over 50 years. The wild-type (C/C) population (n=6) had higher serum UA levels in this group. The average serum UA level was 345.14 μmol/L in the wild type (C/C) population, 298.49 μmol/L in the heterozygous-type (T/C) population (n=55), and 271.20 μmol/L in the mutant (T/T) population (n=168) (P=0.00023). The findings suggested that the presence of C alleles could increase the serum UA level of Chinese Han women aged over 50 years. The mean and standard error of serum UA levels in various genotypes after age stratification is shown in **Table 3**. There were no significant associations between the rs3740165 polymorphism and serum UA levels in all participants or in the group of women aged ≤50 years.

**Table 3:** The mean and standard error of serum uric acid for different genotypes after age stratification.

UA	rs3740165_TT	rs3740165_TC	rs3740165_CC	SE_TT	SE_TC	SE_CC	P-value
All (n=1169)	259.57 (n=878)	263.09 (n=266)	271.53 (n=25)	2.00	3.99	12.81	0.227
≤50 years (n=940)	256.92 (n=710)	253.22 (n=210)	249.13 (n=20)	2.23	3.86	11.81	0.333
>50 years (n=229)	271.20 (n=168)	298.49 (n=55)	345.14 (n=6)	4.43	10.95	23.30	**0.00023**

SE, standard error; UA, uric acid.

**Figure 2:**
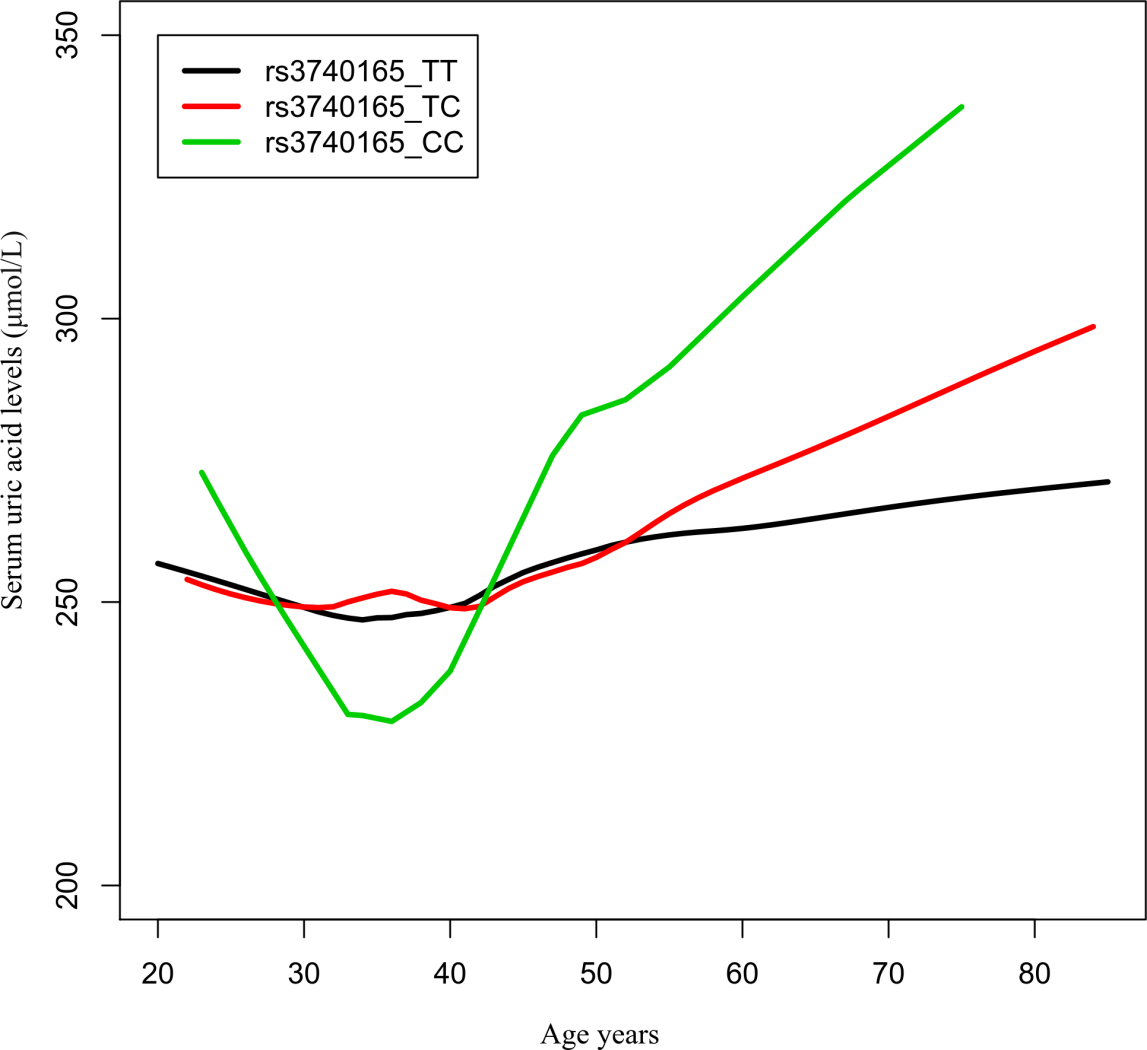
Age−urea acid curves of rs3740165 genotypes. The changes in serum uric acid in three different genotype carriers with age growth.

The number of participants with the rs3740165 genotypes TT, TC, and CC was 878 (75.1%), 266 (22.8%), and 25 (2.1%), respectively. The number of participants with the genotype CC was small; therefore, we grouped the study participants into two groups, the TT group and the TC + CC group, and we used logistic regression to perform further analysis. In the group over 50 years of age, the C allele was associated with a higher risk of hyperuricemia than allele T (odds ratio 2.752, 95% confidence interval 1.458–5.192; P = 0.002). There was also a higher risk of hyperuricemia in genotype TC + CC compared with genotype TT (odds ratio 3.326, 95% confidence interval 1.513–7.313; P = 0.003) in the over 50 years group (**Table 4**). The changes in serum UA in the three different genotype carriers with age growth is presented in **Figure 2**.

**Table 4:** The rs3740165 variants and the risk of hyperuricemia (UA >420 μmol/L).

rs3740165 variants	Odds ratio (95% CI)	P-value
All (n=1169)		
TC + CC vs TT	1.248(0.775–2.009)	0.362
C vs T	1.247(0.822–1.891)	0.299
Age ≤50 years (n=940)		
TC + CC vs TT	0.678 (0.346–1.326)	0.256
C vs T	0.711 (0.385–1.310)	0.274
Age >50 years (n=229)		
TC + CC vs TT	3.326 (1.513–7.313)	**0.003**
C vs T	2.752 (1.458–5.192)	**0.002**

95% CI, 95% confidence interval; UA, uric acid.

## 4 Discussion

The present study investigated the association of 15 candidate genes in the vitamin D metabolic pathway with serum UA levels. The serum UA level was significantly higher in women over 50 years versus women aged ≤50 years. Postmenopausal women have a higher body mass index, higher blood lipid levels, and higher levels of bone turnover markers (33). We observed that genetic variation in the *CUBN* gene is associated with serum UA levels in Chinese Han women over 50 years. Individuals with the C allele of rs3740165 had significantly higher serum UA levels than did T allele carriers. To the best of our knowledge, this is the first study to investigate the common allelic variant in the *CUBN* gene and its association with serum UA levels.

The *CUBN* gene is associated with several diseases. *CUBN* gene mutation may cause Imerslund–Grasbeck syndrome (34, 35) (OMIM 261100), which is a rare autosomal recessive disease characterized by vitamin B12 malabsorption resulting in megaloblastic anemia and asymptomatic proteinuria. In addition, links have been found between *CUBN* and the development and progression of cancers, such as gastric cancer (36), breast cancer (37), renal cell carcinoma (38), and colorectal cancer (39).

The *CUBN* gene is also associated with proteinuria and renal function. For example, human C-terminal *CUBN* variants are associated with chronic proteinuria and normal renal function (40). Ahluwalia et al. identified a rare missense (A1690V) variant in *CUBN* associated with albuminuria in a European meta-analysis (41). Serum UA level is one of the most sensitive indicators of human renal function. No other reports exist concerning the relationship between the *CUBN* genotype and serum UA level.

The SNP in the *CUBN* gene (rs3740165) was identified as being significantly associated with serum UA in women over 50 years, but not in the group aged ≤50 years. This difference may exist for the following reasons. First, quantitative trait loci that regulate serum UA levels in humans may be sex-specific, site-specific, and age-specific. Second, serum UA level is a complex trait, and these are generally controlled by common polymorphisms, resulting in more subtle changes in gene function or expression.

There are genetic factors in urinary serum UA excretion, and several genes are involved in excretion of UA in the urine as well as reabsorption within the kidney; these include *SLC2A9*, *SLC22A12*, *SLC17A1*, *SLC17A3*, and *ABCG2* (13, 16, 42–44). The *GC* gene also has a potential causal association with UA (27). Our findings increase the gene spectrum of serum-UA-related genes and open new avenues for a better understanding of the heritable basis of hyperuricemia.

Our study had the following strengths (1): dense markers and those previously reported in genome-wide association analyses within 15 candidate genes involved in vitamin D metabolism were selected (2); by conducting this study in a healthy population, the analysis of the genetic impact on serum UA levels was not confounded by the potential impact of disease; and (3) in order to avoid the influence of age on serum UA levels, we analyzed the data in groups of women ≤50 years and >50 years.

Nevertheless, the results of our study should be interpreted with the following limitations in mind. First, the main weakness of the study was the small sample group and the difference in the numbers of women aged >50 years and ≤50 years. We should enlarge our sample size in future studies to improve statistical power and to avoid bias. Second, owing to the difficulty in recruiting male participants, the participants in this study were all women. We plan to add male data in order to analyze data from both sexes. Third, UA levels vary across the menstrual cycle (45), but this aspect was not considered in the study. We will unify the menstrual cycle phase when testing the UA level of premenopausal women in future research.

In conclusion, our study provides evidence that genetic variation in the *CUBN* gene is associated with serum UA levels in Chinese Han women over 50 years of age. We suggest that further investigations are conducted including larger sample sizes, male participants, and other ethnic groups.

## Data availability statement

The raw data supporting the conclusions of this article will be made available by the authors, without undue reservation. Requests to access these datasets should be directed to gujiemei81@163.com.

## Ethics statement

The studies involving human participants were reviewed and approved by the Ethics Committee of the Shanghai Jiao Tong University Affiliated Sixth People’s Hospital. The participants provided their written informed consent to participate in this study.

## Author contributions

ZZ designed the study and revised the manuscript. JG collected the blood samples, analyzed the genetic results, did the statistical analysis, and draft the manuscript. HY, CW, HZ, and WH collected clinical information of the participants. All authors read and approved the final manuscript.
