# Supplementary material for: Vitamin D pathway gene variation rs3740165 is associated with serological uric acid levels in healthy Chinese women
Source: Front Endocrinol (Lausanne). 2022 Dec 13;13:1059964. doi: 10.3389/fendo.2022.1059964 (PMC9792855; doi:10.3389/fendo.2022.1059964)
Supplement: Supplementary file 1 [file Table_1.docx]

**Table S1.** The basic characteristics of the SNPs.

|  | Pos(hg19) | Gene | RS | Ref | Alt | Function | HWE | gnomad_AF | our_AF |
| --- | --- | --- | --- | --- | --- | --- | --- | --- | --- |
| 1 | 60358982 | CYP2J2 | rs2280273 | T | C | UTR3 | 0.845 | 0.091 | 0.107 |
| 1 | 60360027 | CYP2J2 | rs1408479 | C | T | intronic | 0.691 | 0.116 | 0.106 |
| 1 | 60373775 | CYP2J2 | rs1155002 | T | C | intronic | 0.510 | 0.718 | 0.729 |
| 1 | 60378853 | CYP2J2 | rs11572254 | G | T | intronic | 0.903 | 0.100 | 0.084 |
| 1 | 60388674 | CYP2J2 | rs10493270 | G | A | intronic | 0.000 | 0.169 | 0.548 |
| 1 | 60389605 | CYP2J2 | rs11572204 | G | C | intronic | 0.158 | 0.141 | 0.129 |
| 10 | 16870912 | CUBN | rs1801232 | G | T | exonic | 0.094 | 0.164 | 0.135 |
| 10 | 16882518 | CUBN | rs703064 | T | C | exonic | 0.017 | 0.478 | 0.513 |
| 10 | 16918972 | CUBN | rs1801241 | A | G | exonic | 0.699 | 0.322 | 0.318 |
| 10 | 16949550 | CUBN | rs3740165 | T | C | exonic | 0.017 | 0.121 | 0.137 |
| 10 | 16979606 | CUBN | rs2356590 | G | T | exonic | 0.955 | 0.184 | 0.208 |
| 10 | 16979661 | CUBN | rs1801234 | T | C | exonic | 0.501 | 0.521 | 0.517 |
| 10 | 16982061 | CUBN | rs2271462 | C | T | exonic | 0.955 | 0.183 | 0.208 |
| 10 | 17008946 | CUBN | rs1687705 | C | G | intronic | 0.739 | 0.322 | 0.341 |
| 10 | 17024503 | CUBN | rs1801231 | G | A | exonic | 0.970 | 0.534 | 0.526 |
| 10 | 17113563 | CUBN | rs1801225 | C | T | exonic | 0.426 | 0.556 | 0.570 |
| 10 | 17140234 | CUBN | rs11254370 | A | T | intronic | 0.139 | 0.686 | 0.669 |
| 10 | 17147521 | CUBN | rs1801224 | G | T | exonic | 0.108 | 0.683 | 0.669 |
| 10 | 17152994 | CUBN | rs1801223 | G | A | exonic | 0.055 | 0.196 | 0.209 |
| 10 | 17156151 | CUBN | rs1801222 | A | G | exonic | 0.527 | 0.804 | 0.797 |
| 10 | 17172952 | CUBN | rs10904881 | C | A | intergenic | 0.338 | 0.422 | 0.374 |
| 10 | 96741426 | CYP2C9 | rs1934967 | C | T | intronic | 0.364 | 0.194 | 0.175 |
| 10 | 96741817 | CYP2C9 | rs1934968 | A | G | intronic | 0.723 | 0.614 | 0.618 |
| 10 | 96746078 | CYP2C9 | rs2298037 | C | T | intronic | 0.358 | 0.299 | 0.338 |
| 10 | 96748495 | CYP2C9 | rs1934969 | A | T | intronic | 0.404 | 0.382 | 0.428 |
| 10 | 124768583 | ACADSB | rs12263012 | G | A | exonic | 0.779 | 0.135 | 0.112 |
| 10 | 124770575 | ACADSB | rs6599641 | T | G | intronic | 0.781 | 0.420 | 0.377 |
| 10 | 124771035 | ACADSB | rs4633392 | C | G | intronic | 0.577 | 0.159 | 0.161 |
| 10 | 124789186 | ACADSB | rs7070793 | A | G | intronic | 0.207 | 0.259 | 0.216 |
| 10 | 124811466 | ACADSB | rs1898462 | C | T | intronic | 0.812 | 0.577 | 0.625 |
| 10 | 124814481 | ACADSB | rs11248371 | A | T | UTR3 | 0.453 | 0.159 | 0.129 |
| 10 | 124818438 | ACADSB | rs7100031 | A | G | downstream | 0.630 | 0.112 | 0.109 |
| 11 | 13514053 | PTH | rs6256 | G | T | exonic | 0.351 | 0.124 | 0.140 |
| 11 | 13514263 | PTH | rs6254 | C | T | intronic | 0.012 | 0.124 | 0.133 |
| 11 | 13514505 | PTH | rs177706 | C | T | intronic | 0.355 | 0.698 | 0.667 |
| 11 | 13516379 | PTH | rs751610 | C | T | intronic | 0.682 | 0.159 | 0.163 |
| 11 | 14909745 | CYP2R1 | rs7936142 | A | T | intronic | 0.364 | 0.172 | 0.130 |
| 11 | 14913575 | CYP2R1 | rs12794714 | G | A | exonic | 0.968 | 0.357 | 0.371 |
| 11 | 14915310 | CYP2R1 | rs2060793 | A | G | intergenic | 0.144 | 0.663 | 0.643 |
| 11 | 14915908 | CYP2R1 | rs16930609 | A | C | intergenic | 0.939 | 0.134 | 0.142 |
| 11 | 14921880 | CYP2R1 | rs10766197 | G | A | intergenic | 0.109 | 0.349 | 0.372 |
| 11 | 71142350 | DHCR7 | rs1790349 | T | C | intergenic | 0.279 | 0.280 | 0.291 |
| 11 | 71144468 | DHCR7 | rs7122671 | G | A | downstream | 0.630 | 0.096 | 0.085 |
| 11 | 71147883 | DHCR7 | rs1790329 | A | G | intronic | 0.542 | 0.786 | 0.835 |
| 11 | 71150296 | DHCR7 | rs1790325 | C | T | intronic | 0.824 | 0.778 | 0.851 |
| 11 | 71154204 | DHCR7 | rs11606033 | A | G | intronic | 0.176 | 0.387 | 0.449 |
| 11 | 71169547 | NADSYN1 | rs2276360 | G | C | exonic | 0.572 | 0.406 | 0.463 |
| 11 | 71174553 | NADSYN1 | rs1629220 | C | T | intronic | 0.936 | 0.370 | 0.379 |
| 11 | 71183737 | NADSYN1 | rs2282618 | T | C | intronic | 0.927 | 0.835 | 0.885 |
| 11 | 71210023 | NADSYN1 | rs10898210 | G | A | intronic | 0.754 | 0.758 | 0.816 |
| 11 | 71211654 | NADSYN1 | rs4944076 | G | A | intronic | 0.757 | 0.700 | 0.764 |
| 11 | 71212219 | NADSYN1 | rs12277408 | T | C | intronic | 0.543 | 0.137 | 0.123 |
| 12 | 58162085 | CYP27B1 | rs10877012 | G | T | downstream | 0.716 | 0.624 | 0.639 |
| 12 | 58162739 | CYP27B1 | rs703842 | A | G | UTR3 | 0.572 | 0.632 | 0.639 |
| 15 | 74634899 | CYP11A1 | rs11638442 | C | G | intronic | 0.575 | 0.204 | 0.240 |
| 15 | 74640109 | CYP11A1 | rs1484215 | C | T | intronic | 0.056 | 0.188 | 0.177 |
| 15 | 74655583 | CYP11A1 | rs7174179 | A | G | intronic | 0.492 | 0.560 | 0.572 |
| 15 | 74661894 | CYP11A1 | rs2073475 | C | T | intergenic | 0.024 | 0.456 | 0.416 |
| 15 | 74662387 | CYP11A1 | rs16968477 | C | T | intergenic | 0.380 | 0.309 | 0.306 |
| 15 | 75012985 | CYP1A1 | rs1048943 | T | C | exonic | 0.759 | 0.251 | 0.239 |
| 15 | 75015305 | CYP1A1 | rs4646422 | C | T | exonic | 0.014 | 0.150 | 0.170 |
| 15 | 75016192 | CYP1A1 | rs4646421 | G | A | intronic | 0.083 | 0.407 | 0.390 |
| 2 | 219651819 | CYP27A1 | rs6716642 | G | A | intronic | 0.509 | 0.078 | 0.076 |
| 2 | 219663153 | CYP27A1 | rs6740004 | G | A | intronic | 0.508 | 0.160 | 0.169 |
| 20 | 52771171 | CYP24A1 | rs4809957 | A | G | UTR3 | 0.127 | 0.636 | 0.635 |
| 20 | 52772741 | CYP24A1 | rs927650 | T | C | intronic | 0.073 | 0.741 | 0.739 |
| 20 | 52773510 | CYP24A1 | rs6097809 | T | C | intronic | 0.379 | 0.328 | 0.307 |
| 20 | 52778961 | CYP24A1 | rs2209314 | T | C | intronic | 0.702 | 0.428 | 0.415 |
| 20 | 52781251 | CYP24A1 | rs2762939 | G | C | intronic | 0.462 | 0.282 | 0.285 |
| 20 | 52781264 | CYP24A1 | rs6013905 | T | C | intronic | 0.693 | 0.358 | 0.379 |
| 20 | 52782691 | CYP24A1 | rs3787555 | C | A | intronic | 0.339 | 0.584 | 0.581 |
| 20 | 52783135 | CYP24A1 | rs3787557 | T | C | intronic | 0.445 | 0.263 | 0.242 |
| 20 | 52783652 | CYP24A1 | rs2762941 | G | A | intronic | 0.286 | 0.619 | 0.649 |
| 20 | 52784478 | CYP24A1 | rs2181874 | G | A | intronic | 0.809 | 0.255 | 0.259 |
| 20 | 52785859 | CYP24A1 | rs4809959 | A | G | intronic | 0.348 | 0.440 | 0.452 |
| 20 | 52786219 | CYP24A1 | rs2296241 | G | A | exonic | 0.247 | 0.407 | 0.411 |
| 20 | 52788013 | CYP24A1 | rs6022999 | A | G | intronic | 0.720 | 0.217 | 0.240 |
| 20 | 52791518 | CYP24A1 | rs2248359 | C | T | intergenic | 0.780 | 0.375 | 0.375 |
| 4 | 72601331 | GC | rs12512631 | T | C | intergenic | 0.029 | 0.205 | 0.176 |
| 4 | 72608115 | GC | rs705117 | C | T | intronic | 0.527 | 0.490 | 0.513 |
| 4 | 72608383 | GC | rs2282679 | T | G | intronic | 0.547 | 0.256 | 0.319 |
| 4 | 72609094 | GC | rs1491710 | A | C | intronic | 0.047 | 0.338 | 0.313 |
| 4 | 72611690 | GC | rs842999 | G | C | intronic | 0.313 | 0.233 | 0.196 |
| 4 | 72614140 | GC | rs705120 | A | C | intronic | 0.911 | 0.557 | 0.502 |
| 4 | 72616932 | GC | rs222040 | G | A | intronic | 0.449 | 0.362 | 0.330 |
| 4 | 72618334 | GC | rs7041 | A | C | exonic | 0.271 | 0.295 | 0.266 |
| 4 | 72624135 | GC | rs10488854 | C | T | intronic | 0.752 | 0.207 | 0.180 |
| 4 | 72631744 | GC | rs222010 | T | C | intronic | 0.296 | 0.692 | 0.731 |
| 4 | 72636272 | GC | rs222020 | C | T | intronic | 0.437 | 0.597 | 0.375 |
| 4 | 72643488 | GC | rs1155563 | T | C | intronic | 0.586 | 0.334 | 0.412 |
| 4 | 72644962 | GC | rs222029 | G | A | intronic | 0.719 | 0.780 | 0.808 |
| 4 | 72647762 | GC | rs1352845 | A | G | intronic | 0.134 | 0.400 | 0.337 |
| 7 | 99354114 | CYP3A4 | rs12333983 | T | A | downstream | 0.469 | 0.301 | 0.290 |
| 7 | 99361466 | CYP3A4 | rs2242480 | C | T | intronic | 0.960 | 0.286 | 0.247 |
| 7 | 99365083 | CYP3A4 | rs4646437 | G | A | intronic | 0.000 | 0.161 | 0.113 |
